# A rare case of hyalinizing clear cell carcinoma of the tongue root: A case report and literature review

**DOI:** 10.3892/ol.2025.14914

**Published:** 2025-02-04

**Authors:** Langqing Liu, Xue Cao, Yingjie Xu, Jie Wang, Xiao Tian, Yulian Fu, Bin Ling

**Affiliations:** 1 Department of Oral-Maxillofacial Oncology and Surgery, The First Affiliated Hospital of Xinjiang Medical University, Urumqi, Xinjiang Uyghur Autonomous Region 830000, P.R. China; 2 School/Hospital of Stomatology, Xinjiang Medical University, Urumqi, Xinjiang Uyghur Autonomous Region 830000, P.R. China; 3 Stomatological Research Institute of Xinjiang Uyghur Autonomous Region, Urumqi, Xinjiang Uyghur Autonomous Region 830000, P.R. China

**Keywords:** HCCC, tongue, treatment, prognosis

## Abstract

The present study describes a rare case of hyalinizing clear cell carcinoma (HCCC) of the tongue root and provides an analysis and review of the relevant literature to improve the understanding of its diagnosis and treatment. Clinical imaging and pathological data from a patient with primary HCCC of the tongue root were summarized, and previously published studies were reviewed through a literature search. The common symptoms, treatment strategies and prognoses reported in the literature were compared. A total of 16 cases of primary HCCC of the tongue were retrieved. Histologically, these cases had tumors characterized by clear cells arranged in sheets, nests and cords within the fibrous interstitium surrounding tumor cells, with round to oval cell nuclei occasionally containing small or inconspicuous nucleoli. Immunohistochemistry showed positive tumor cell staining for cytokeratin (CK)5/6, CK7 and p63, and negative for S-100, smooth muscle actin and calponin. Clinical symptoms included dysphagia, a painless mass, tongue root ulceration and a foreign body sensation in the throat. Treatment strategies included surgery, radiation therapy, repair and reconstruction, with no local recurrence or metastasis at any follow-up point. The present findings indicated that HCCC of the salivary glands is an inert malignant tumor, and a good prognosis can be achieved with both surgical resection and radiation therapy.

## Introduction

Hyalinizing clear cell carcinoma (HCCC), also known as clear cell carcinoma with hyalinization, is a rare, low-grade malignant neoplasm originating from the minor salivary glands. Initially described by Batsakis (1) in 1980, the definition of HCCC was later refined by Simpson *et al* (2) and Milchgrub *et al* (3). HCCC has been referred to as clear cell adenocarcinoma, clear cell carcinoma (nonspecific) or clear cell carcinoma by various authoritative sources, including the Pathology Atlas Volume of the Military Forces Institute of Pathology (4), the 3rd edition of the World Health Organization (WHO) Head and Neck Tumor Pathology and Genetics Classification (5), and the 4th edition of the WHO Head and Neck Tumor Classification (6). In the 5th edition of the WHO Classification of Head and Neck Tumors in 2022 it was renamed HCCC (7).

Salivary gland tumors account for 0.5% of all malignant tumors, with clear cell carcinoma of the salivary gland representing ~1% of all salivary gland tumors globally. HCCC occurs only in the minor salivary glands and is characterized by slow growth, presenting as small inert masses with non-aggressive biological behavior (8). Morphologically, HCCC often presents as an irregularly shaped, hard mass with a rough, grayish-white and grayish-red surface. The tumor appears grayish-white in histological analysis, with hemorrhage and necrosis commonly seen in the center. The tumors exhibit poorly defined boundaries and infiltrate the surrounding tissues, with diameters typically ranging from 1 to 5 cm. A study in China involving 10 patients with clear cell carcinoma of the salivary gland reported tumor diameters measuring 1.5-5.0 cm, with a mean of 3 cm (9). Additionally, Zhang *et al* (10) analyzed the histological morphology of eight cases diagnosed as HCCC of the salivary gland at the Department of Pathology, The Affiliated Cancer Hospital of Fudan University between January 2015 and October 2019. A basal cell-like arrangement was seen in a few cases, with occasional keratinization in the nests. This previous study concluded that, histologically, the tumor showed infiltrative growth, and the tumor cells were arranged in trabecular, cord-like or solid nest structures.

Because of its rarity, HCCC lacks sufficient clinical trials to establish standardized treatment protocols. Moreover, it is not well known to pathologists, leading to frequent misdiagnoses. Therefore, the present study aimed to provide a comprehensive understanding of clear cell carcinoma of the salivary glands from various perspectives, including clinical signs, imaging features, pathological manifestations, treatment methods and prognosis, through a case report and literature review.

## Case report

### Clinical data and medical history

The patient was a 52-year-old woman who presented with a foreign body sensation at the root of the tongue and dysphagia for >1 month. The patient denied experiencing pain, dyspnea, voice changes or any generalized discomfort, and reported no history of smoking or alcoholism. No enlarged lymph nodes were detected in the bilateral maxillofacial area and neck. The tongue mobility was fair, with centered extension, and no apparent signs of enlargement were observed at the root of the tongue.

A total of 2 weeks before admission, the patient visited the otorhinolaryngology department of a local hospital, where a laryngoscopy revealed a new mass at the root of the tongue. The excised mass was then subjected to a biopsy, and the pathology results indicated a malignant tumor of small salivary gland origin, with a high likelihood of mucoepidermoid carcinoma (MEC).

### Preoperative imaging

**Figure 1.**
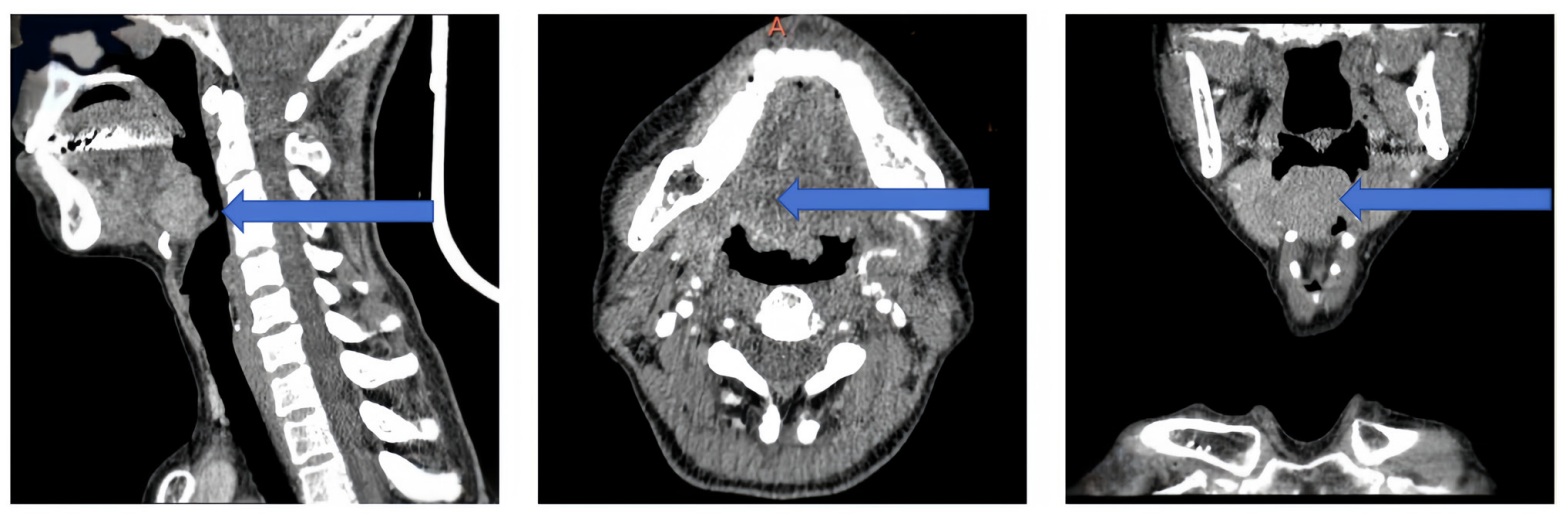
Preoperative enhanced CT images of the patient. The sagittal, horizontal and coronal planes of the CT images reveal a well-defined nodular soft tissue shadow on the right side of the tongue root (arrow). The lesion had regular edges, although the tongue border was unclear. The maximum cross-section measured ~3.7×2.3 cm, with no enlarged lymph nodes in the maxillofacial and cervical regions. Additionally, there was no apparent thickening or protrusion on either side of the inner wall of the vocal folds. The laryngeal soft tissue structure remained symmetrical, with no apparent high-density masses. The epiglottis was not visibly enlarged, the pyriform sinuses on both sides and the paralaryngeal space were clear, and there was no damage to the surrounding bone. CT, computed tomography.

**Figure 2.**
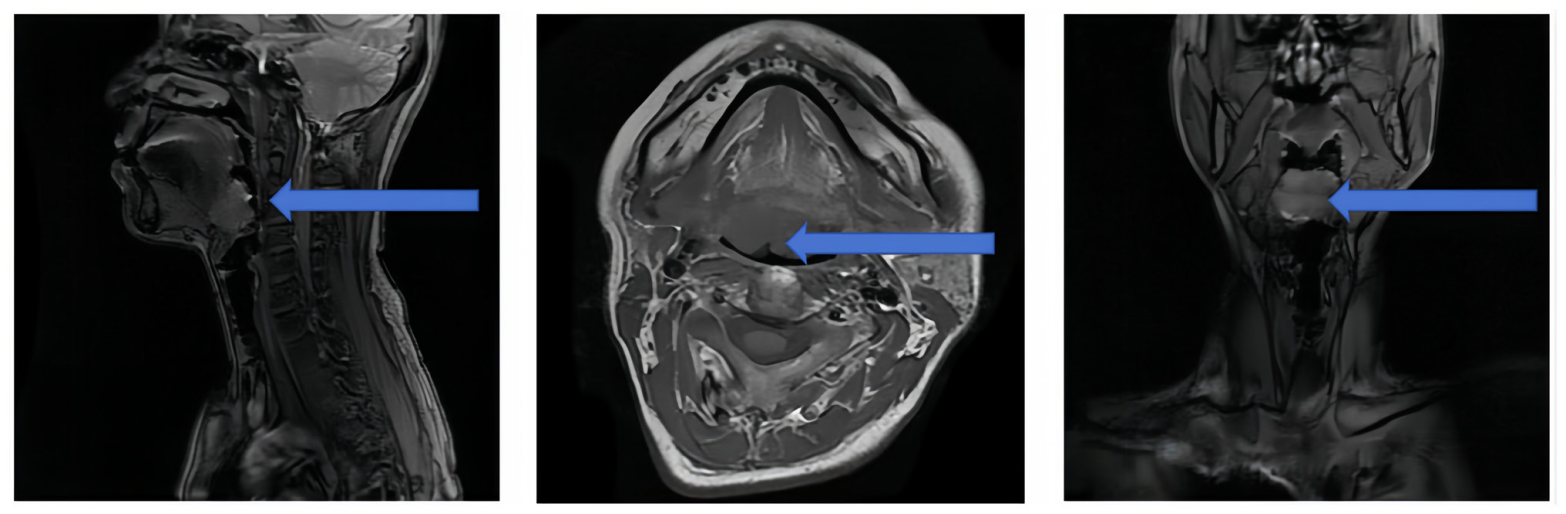
Preoperative laryngeal magnetic resonance imaging of the patient. The image revealed a soft tissue mass shadow on the right side of the posterior part of the tongue root with slightly high signal intensity on the T2WI fat-compression image (arrow), along with a linear isointense shadow within the lesion. The lesion measured ~3.5×2.3 cm, with compression of the epiglottic vallecula. The enhancement scan showed significant homogeneous lesion enhancement and no obviously enlarged lymph nodes were observed bilaterally in the neck.

Enhanced head and neck computed tomography (CT) revealed a homogeneously enhancing mass to the right of the tongue root, causing narrowing of the right epiglottic vallecular (Fig. 1). Magnetic resonance imaging (MRI) of the head and neck showed an abnormal signal to the right of the posterior root of the tongue, suggesting an abnormal localization (Fig. 2).

### Preoperative pathology result

Immunohistochemical staining revealed the following results: p40 (+), cytokeratin (CK)5/6 (+), p53 (+50%), CK7 (+) and P16 showing partial positivity. Following consultation with the local hospital pathology department, the diagnosis was refined to a salivary gland epithelial tumor with low malignancy.

### Surgical procedure

The treatment plan and potential surgical complications were discussed with the patient and their family, and an informed consent form was signed. The procedure involved a right functional neck dissection, localized extended lumpectomy of the right tongue root, median mandibulotomy and anterolateral thigh flap transplantation.

A post-cervical lymphatic incision was made on the right side of the neck, allowing for clearance of the right functional cervical lymph nodes. The internal jugular vein and parasympathetic nerves were preserved and the tongue root and mass were fully exposed through the median mandibular splitting. Following the No-Tumor Principle (11), a partially enlarged surgical resection was performed at the edge of the mass, and an anterolateral thigh flap was used to repair the defective area of the tongue root. Postoperatively, a tracheostomy was performed, and the right side of the tongue root mass and the lymphatic tissue were histopathologically examined.

### Postoperative pathological results

**Figure 3.**
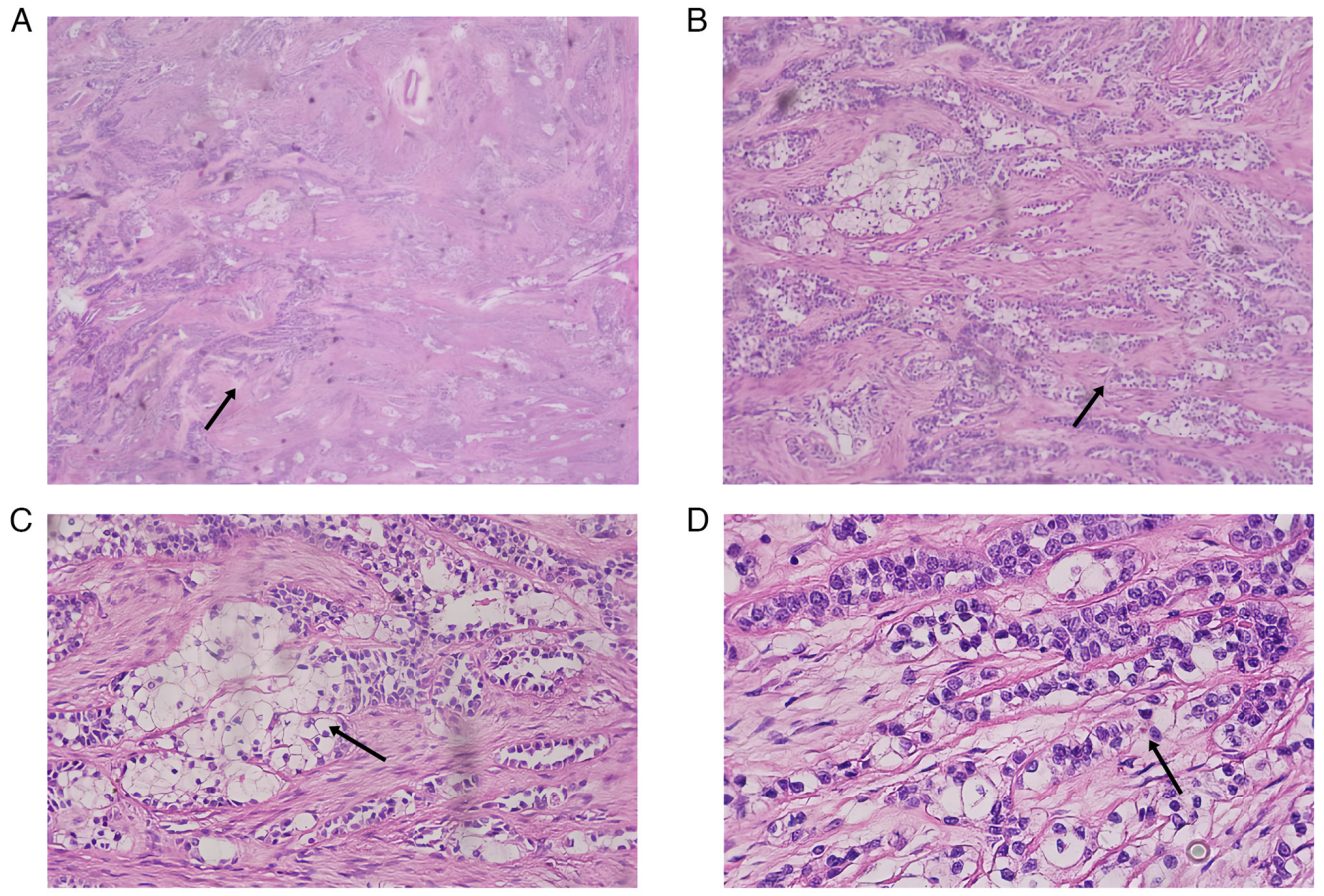
Hematoxylin and eosin staining results of the tongue root mass. (A) The area indicated by the arrow shows the cell nuclei were small with inconspicuous nucleoli and distinct cell boundaries (magnification, ×40). (B) Hyaline cells formed strips or trabeculae, extruding hyalinized and sclerotic fibrous mesenchyme (arrow) (magnification, ×100). (C) The stroma surrounding the cell nests shows a fibrillary red staining, and the mesenchyme around the tumor was fibrocystic in nature. Tumor cells infiltrated into the fibrous interstitium as indicated by the arrow (magnification, ×200). (D) Cancer cells were distributed in nests within the fibrous interstitium with a more homogeneous nuclear morphology, and the phenomenon of nuclear fission was infrequent as indicated by the arrow (magnification, ×400).

Histopathologically examining the tongue root mass revealed mucosal irregularities, measuring 6.2×5.4×3.8 cm, with grayish-white nodules. The tumor, measuring 4.2×2.3×2 cm, exhibited a relatively firm consistency. Hematoxylin and eosin staining revealed that the tumor cells were arranged in sheets, nests and thin cords. The cytoplasm of the tumor cells was transparent, the stroma around the nests of the cells was reddish-stained, and the mesenchymal stroma around the tumor showed fibrous changes (Fig. 3). The fixative was 10% formalin and the tissues were fixed at room temperature for 24 h. The thickness of the sections was 4 µm. Staining was performed at room temperature, with Hematoxylin applied for 5 min and Eosin for 2 min. We used a Nikon Eclipse Ti2 inverted microscope.

**Figure 4.**
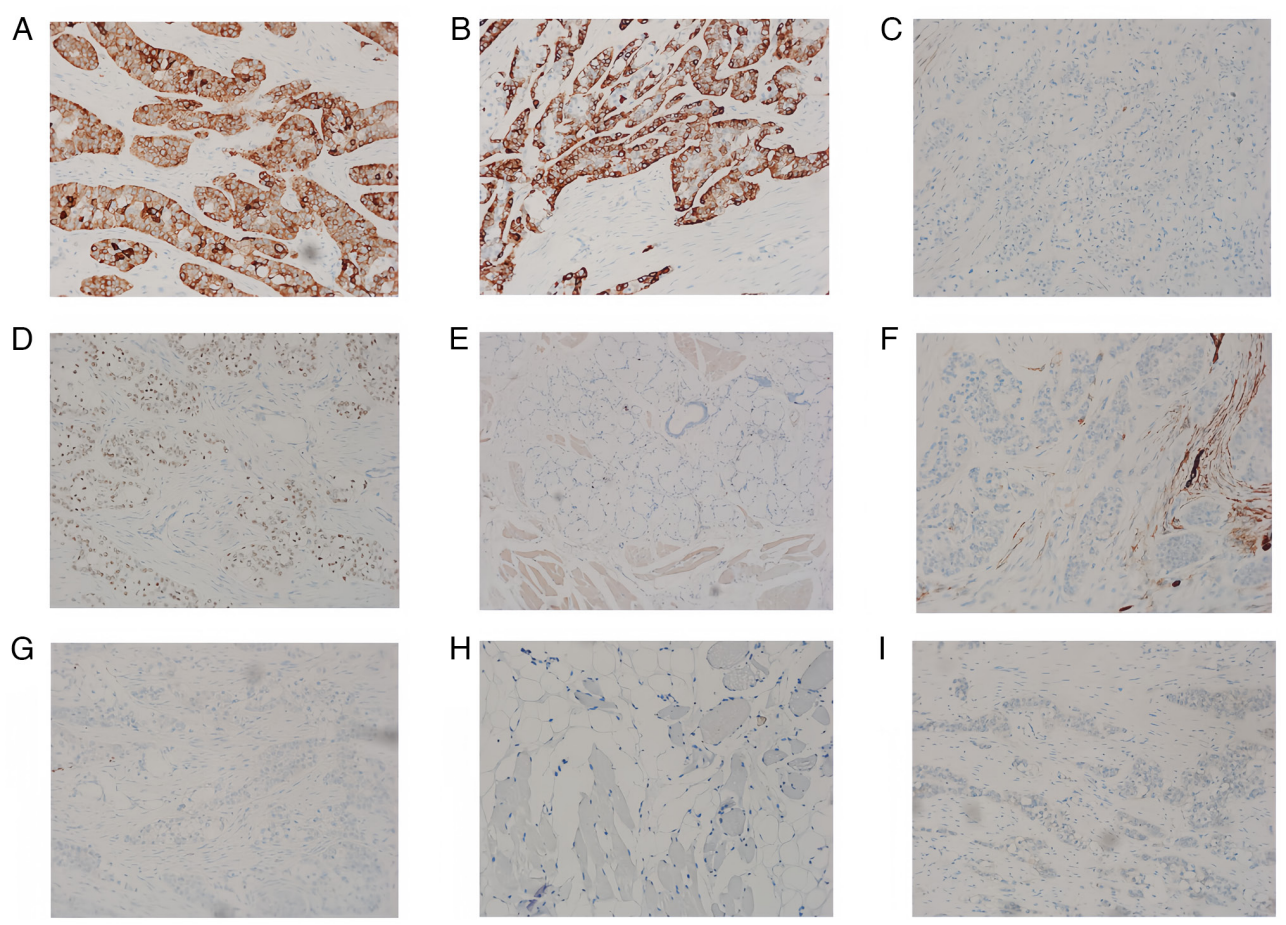
Immunohistochemical staining results of the tongue root mass. (A) CK5/6 (+), magnification ×100, (B) CK7 (+), magnification ×100, (C) calponin (−), magnification ×40, (D) p63 (+), magnification ×40, (E) Ki-67 (5%+), magnification ×40, (F) CD34 (−), magnification ×40, (G) S-100 (−), magnification ×40, (H) SOX10 (−), magnification ×40 and (I) DOG-1 (−), magnification ×40. CK, cytokeratin.

Immunohistochemical staining revealed the following results: CK5/6 (+), CK7 (+), calponin (−), p63 (+), CD117 (−), p40 (+), Ki-67 (5% +), CD34 (−), S-100 (−) and SOX10 (−; Fig. 4). The tissues used for immunohistochemistry were paraffin-embedded, and the sections were cut to a thickness of 4 micrometers. The blocking reagent used was 5% BSA (Thermo Fisher Scientific, Inc.), applied at room temperature for 1 h. The primary antibody was diluted to 1:200, obtained from Roche, America, catalogue number CK5/6: 790-4554; CK7: 790-4462; Calponin: 760-4376; P63: 790-4509; CD117: 08763909001; P40: 790-4950; Ki-67: 790-4286; CD34: 790-2927; SOX10: 790-4968; DOG-1: 760-4590; S-100: 790-2914, and incubated overnight at 4°C. Secondary antibody dilution: 1:200, catalogue number: bs-9912R, supplier, conjugate, Bioss, China, temperature: 25°C and duration of incubation: 1 h. The images were captured using Nikon Eclipse Ti2 inverted microscope. Although acinic cell carcinoma (AciCC) and squamous cell carcinoma of the head and neck were suspected, they were ruled out by a negative DOG1 result (Fig. 4) (12). DOG1, or discovered on gastrointestinal stromal tumors 1, is an immunohistochemical marker primarily used to identify AciCC among salivary gland tumors, as AciCC often exhibits positive DOG1 expression. According to Khurram and Speight (12), DOG1 is valuable for differentiating AciCC from tumors with similar histological features, such as clear cell carcinoma, where DOG1 is typically negative. In the present case, the negative DOG1 result helped exclude AciCC, guiding toward a rarer diagnosis of HCCC of the tongue root. Fluorescence *in situ* hybridization (FISH) results revealed positive breakage recombination of the *EWSR1* gene (Fig. S1). Based on these findings, the morphology and immunophenotype of the mass were indicative of clear cell carcinoma of the salivary gland. No metastasis was observed in the cervical lymph nodes (0/5).

After consulting with the multidisciplinary tumor team and considering the extensive nature of the surgery, the patient received 1 month of adjuvant radiation therapy. The specific radiation therapy plan, based on the diagnosis of T3N0M0 clear cell carcinoma at the tongue root, outlined the target area as the preoperative tumor area and bilateral cervical lymph nodes (areas Ib, II and III). A total dose of 60 Gy was administered over 30 routine irradiation sessions, with each session delivering 2 Gy, conducted 6 times/week for 5 weeks. Because it was a low-grade malignant tumor, radiotherapy was not planned for the lymph node drainage area in zone IV. The target area, including the oropharyngeal mucosa, was relatively large, and the patient exhibited a slightly heightened radiation response.

### Postoperative imaging examination

**Figure 5.**
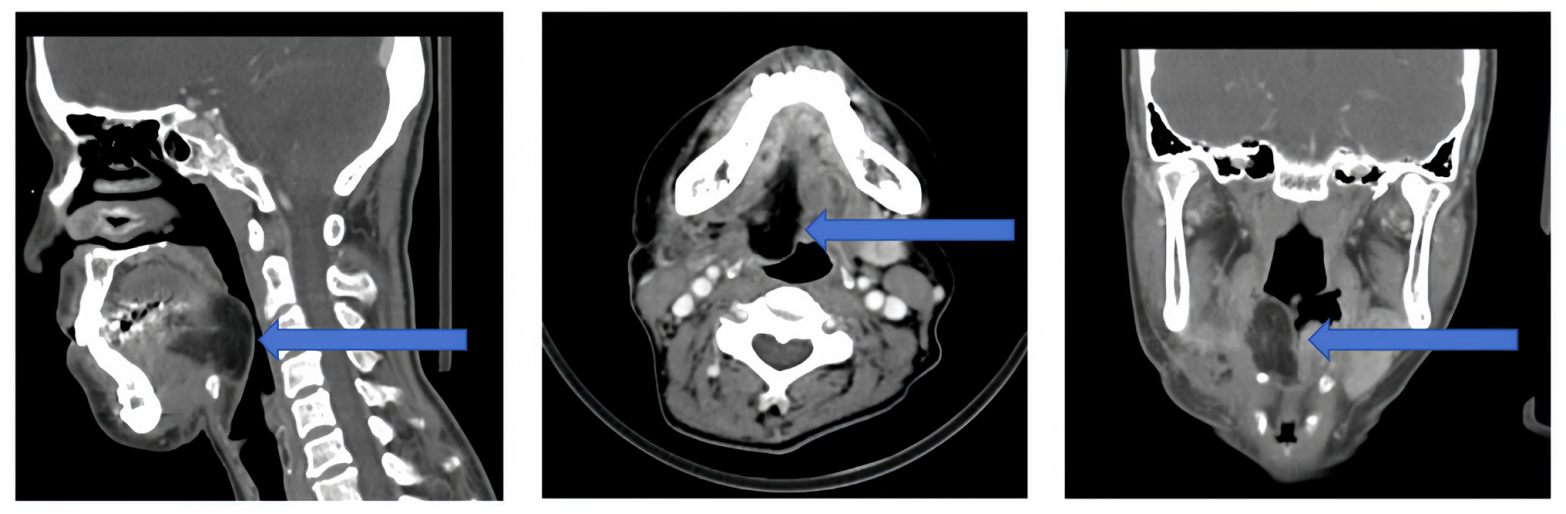
Postoperative enhanced computed tomography of the tongue. The image revealed discontinuity in the alignment of the cortical and mandibular bones. The right side of the tongue was partially missing, and the structure of the operative area was disorganized (arrow). The right submandibular gland was not visible, and the operative area appeared as a dense nodular shadow. Pore patches of adipose tissue accumulation and soft tissue density shadows were observed from the operative area to the right sternocleidomastoid muscle. The enhancement scan of the operative area showed linear enhancement of density shadows. The right submandibular area of soft tissue was slightly swollen, and the surrounding fat interstitial space was slightly turbid. The right laryngopharyngeal and oropharyngeal cavities were slightly narrowed, indicative of postoperative change.

A total of 2 months following the operation, an enhanced CT scan of the tongue was performed. The results showed the structural disorder of the tongue root and disorganization of the mandibular operation area. Mixed-density and linear enhancement from the operative area to the right sternocleidomastoid muscle tract were also observed. The right submandibular gland was not visible (Fig. 5), consistent with the postoperative changes from the localized surgery. There were no apparent signs of recurrence or metastasis.

### Medical history

The patient underwent a biopsy in August 2023, at the People's Hospital of Changji Hui Autonomous State (Xinjiang Uyghur Autonomous Region, China. They were admitted to The First Affiliated Hospital of Xinjiang Medical University (Urumqi, China) 7 days later and were treated by a surgeon 5 days after admission. The patient was discharged in September 2023. The patient was followed up at 1 month, three and six months after discharge, with the most recent follow-up in July 2024. The preoperative immunohistochemistry and biopsy pathology images are unavailable, as these diagnostic procedures were performed at another hospital (People's Hospital of Changji Hui Autonomous State).

### Literature review

**Figure 6.**
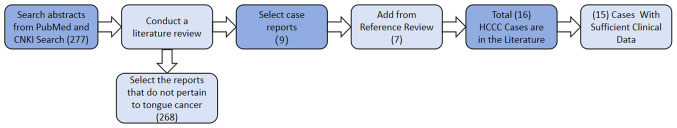
Schematic diagram of the literature review for HCCC. HCCC, hyalinizing clear cell carcinoma.

A comprehensive literature review was performed by searching the key words ‘clear cell carcinoma’ and ‘salivary gland’ in the PubMed (pubmed.ncbi.nlm.nih.gov) and CNKI (cnki.net) databases. After excluding reports on clear cell carcinoma in non-salivary gland areas and non-tongue primary lesions, 277 reports were retrieved. After thoroughly reviewing these reports and their relevant references, 15 reports with adequate clinical data, including complete pre- and postoperative information, were identified (Fig. 6).

Table I presents the clinical and statistical features of 16 cases of primary clear cell carcinoma of the tongue, including the present case (13–25). Among these patients, nine were female and seven were male, with an average age of 53 years (range: 33-69 years). Typical symptoms included dysphagia, a painless mass, tongue root ulceration and a foreign body sensation in the throat. Dysphagia occurred in eight cases and a painless mass was noted in three cases, with the duration of these symptoms ranging from 1 to 6 months. The tumor size was mentioned in 11 cases, with an average diameter of 3.27 cm (range: 1.00-5.50 cm). The primary tumor lesion was located in the tongue in all 16 patients, with 14 in the tongue root and two in the ventral tongue. Lymph node metastasis was observed in five patients.

Treatment was consistent across cases, with all patients undergoing extended local tumor resection. Additionally, seven of the 16 patients underwent repair and reconstruction, and five received postoperative radiotherapy. Postoperative follow-up information was unavailable for two patients; however, no recurrence was observed in the other 14 patients during the follow-up period. The findings suggested that clear cell carcinoma has a good prognosis when treated with localized mass-enlarged resection and postoperative adjuvant radiotherapy.

## Discussion

HCCC of the salivary glands typically presents as a slow-growing, painless submucosal mass with no surface ulceration. Consequently, symptoms are often present for an extended period before the patient seeks treatment. Most of the aforementioned cases involve tumors with a size of 3-5 cm in diameter (13–25).

Clear cells are present in a number of other salivary gland tumors, necessitating differential diagnoses that rely on a combination of immunohistochemistry, specific staining and the morphological features of HCCC of the salivary glands. The histological features observed in the 16 cases reported on in the present study were as follows: Tumor cells were arranged in sheets, nests or thin cords with clear boundaries; the cytoplasm was transparent; the nuclei were round or oval in shape and relatively uniform in size; the nucleoli were inconspicuous; and mitotic figures were rare. In addition, nuclear fission was rare; the stroma around the cell nests was stained red, and the mesenchyme around the tumor was fibrous. The tumor cells grew infiltratively into the fibrous mesenchyme. Immunohistochemical results were positive for epithelial markers, such as CK5/6, CK7 and p63, and negative for myoepithelial markers, such as S-100 and SOX-10.

The immunohistochemical features of HCCC of the salivary gland overlap with those of various salivary tumors, such as MEC and squamous cell carcinoma, all of which are positive for CK7, p63 and p40, and negativity for S-100 and SOX-10. In the last decade, advances in molecular techniques have demonstrated recurrent genetic alterations in some salivary gland tumors, including the fusion of genes such as *ETV6* in secretory carcinoma, *MYB* and *MYBL1* in adenoid cystic carcinoma, and *MAML2* in MEC (26–28). Additionally, *EWSR1*-*ATF1* rearrangements have been found in HCCC, and *HRAS* exon three mutations are seen in most cases of epithelial-myoepithelial carcinoma (29,30). *HRAS* exon three mutations and a high percentage of *EWSR1* rearrangements are commonly detected in clear cell subtype myoepithelial carcinoma (31). FISH technology serves a vital role in pathological research, particularly in detecting recombination in the *EWSR1* gene, a significant genetic alteration commonly observed across various tumor types (32–34). FISH allows for the precise detection of *EWSR1* gene recombination, aiding in tumor characterization. The technique has high sensitivity and specificity for identifying chromosomal abnormalities, making it an integral part of diagnostic processes (35–37). In the present case, FISH results showed positive *EWSR1* gene breakage recombination, confirming the diagnosis of HCCC of the salivary glands and ruling out MEC.

HCCC can be differentiated from MEC and metastatic clear cell carcinoma (MCCC) in several ways (31,38). First, MEC is a malignant tumor with varying proportions of mucous, intermediate and epidermoid cells. It can occasionally include columnar cells, clear cells and eosinophils. While the tumor often demonstrates cystic growth, a clear cell component is generally rare and atypical. Second, the most common origin site of clear cell carcinoma is the kidney, and thus, MCCC typically arises from distant organs, such as the kidneys. Clinically, MCCC presentation varies depending on the site of metastasis. Imaging studies such as CT, MRI and pathological evaluations, including immunohistochemical staining, are crucial for an accurate diagnosis. Immunohistochemistry of MCCC typically shows positivity for PAX8 and CK7, along with increased expression of HIF-1α and VEGF. The pathological features of MCCC resemble those of primary clear cell carcinoma, but a thorough medical history, imaging and specific immunohistochemical markers can help make a proper differentiation. Based on these differences and the tumor origin, the present study ruled out a diagnosis of either MEC or MCCC.

Due to the rarity of HCCC in the salivary glands, there are insufficient clinical trials to determine standardized treatment protocols. Most malignant salivary gland tumors require postoperative radiation therapy to reduce the recurrence rate owing to undesirable features, such as limited margins for resection. Postoperative radiation therapy is also indicated for some moderately to highly differentiated tumors with T-stage 3-4 or lymph node metastases (39). All 16 cases of primary HCCC of the tongue assessed in the present literature review underwent localized enlarged mass resection; five cases underwent postoperative radiotherapy, whereas 11 did not. None of the patients experienced local recurrence or lymph node metastasis during the follow-up period. Desai *et al* (40) specifically analyzed 201 of 254 cases of HCCC of the salivary glands and described the treatment options. The most common approach was surgical resection with extensive margins (81.1%). Cervical lymph node dissection was performed in 10.4% of the cases. Adjuvant treatments were rarely performed, with radiotherapy or chemotherapy administered in only 17.9% of the cases. Of the 223 cases in which recurrence was reported, at least one localized recurrence was observed in 15.2% of cases and more than one recurrence in 3.6%, resulting in a recurrence rate of 18.8% (40). Analyses of the salivary gland cases collected by Desai *et al* (40), along with the cases of primary HCCC of the tongue collected in the present study, showed a low recurrence rate, likely due to the low degree of malignancy, low biological aggressiveness, and low rate of lymphatic and distant metastasis of the tumors. It was also indicated that patients with HCCC of the salivary gland had a better overall prognosis if they underwent complete localized extended resection with or without postoperative radiotherapy. However, despite the low malignancy and recurrence rate of this type of cancer, lymph node, lung and spinal metastases have been reported in a number of cases (38,40–43). Therefore, long-term clinical follow-up after complete tumor resection is essential.

The present study has one specific limitation; photographic documentation of the surgical specimen was not obtained during the procedure. However, detailed written records and descriptions were meticulously maintained to ensure comprehensive case documentation.

In conclusion, HCCC is a rare, low-grade malignant salivary gland tumor characterized by slow clinical progression. It is often confused with benign or other salivary gland tumors, and its diagnosis relies on complete histological morphology and immunohistochemical examination. The FISH test for the fusion of the *MAML2* and *EWSR1* genes aids in making a conclusive diagnosis. The preferred treatment is extended resection of the localized mass, with radiotherapy based on lymph node metastasis and pathological examination to minimize local recurrence and improve the overall patient prognosis.

There are relatively few reports of HCCC occurring in the maxillofacial region, and the present case provides some new insights into its diagnosis and treatment. It is necessary to build a solid foundation to enhance knowledge of this disease, including its clinical manifestations, imaging features and treatment options. This will improve differential diagnosis for this rare disease when patients present with these characteristics. Surgical treatment of HCCC should be specialized and distinct from standard procedures. Future research may delve more deeply into the molecular and genetic mechanisms underlying HCCC.

## Supplementary Material

Supporting Data

## Figures and Tables

**Table I.** Published cases of primary clear cell carcinoma of the tongue root.

First author, year	Age, years/sex	Clinical symptoms	Tumor size and location	Metastasis	Treatment	Follow-up	Results	(Refs.)
Present case	52/F	Foreign body	4.2×2.3×2.0 cm;	No metastasis	The right-side	No recurrence	CK5/6 (+), CK7 (+),	-
		sensation at the	located on the		functional	within 3 months	calponin (−), p63 (+),	
		tongue root	right side behind		neck dissection +		CD117 (−), p40 (+),	
		with dysphagia	the tongue root		the right side of		Ki-67 (5% +),	
		for 1 month			the tongue root		CD34 (−), S-100 (−),	
					local extended		SOX10 (−) and	
					lumpectomy +		DOG-1 (−); positive	
					median		breakage recom-	
					mandibulotomy +		bination of the	
					Anterolateral		*EWSR1* gene	
					thigh flap			
					transplantation +			
					tracheostomy +			
					postoperative			
					radiotherap			
Dabas *et al*, 2023	33/F	Dysphagia, voice	5.5×4.4×4.1 cm;	Not mentioned	Surgical excision +	No recurrence	Not mentioned	(13)
		changes and right	located bilaterally		tracheostomy +	as of 2023		
		ear pain for	at the tongue root,		postoperative			
		6 months	extending to the		radiotherapy			
			anterior tongue,					
			adjacent to the					
			epiglottis and					
			tonsils					
Sento *et al*, 2020	59/M	A painless mass	2.8×2.1×1.5 cm;	Not mentioned	Complete tumor	No local	p63 (+), S-100 (−),	(14)
		on the inferior	located on the		resection + partial	recurrence or	αSMA (−), CD10 (−),	
		surface of the	inferior surface		glossectomy with	metastasis at	GFAP (−),	
		tongue	of the tongue		~1 cm safe margin +	the 5-year	vimentin-Ki-67	
					reconstruction with	postoperative	<10% and	
					a free forearm flap	follow-up	*EWSR1-ATF1* fusion	
Yoldez *et al*, 2022	37/M	An ulcerated and	3.0×0.5 cm;	Not mentioned	Surgical excision	Not mentioned	p40 (+), CD10 (−)	(15)
		painful indu-	located at the				p40 (+), CD10 (−)	
		ration of the base	base of the tongue;					
		of the tongue	poorly circum-					
			scribed with					
			infiltration of the					
			adjacent tissue					
Pillai *et al*, 2019	42/M	Swallowing diffi-	3.0×2.0 cm;a large,	No neck nodes	Excision biopsy +	No local	CK AE1/AE3 (+),	(16)
		culty for 5 months	smooth, broad-	were palpable;	coblation +	recurrence at	CK5/6 (+), p63 (+),	
		and feeling of	based mass at the	no metastasis	postoperative	1-year	S-100 (−) and	
		choking sensation	posterior one-third		radiotherapy	follow-up	CD10 (−)	
		in the throat	of the tongue					
		intermittently.	obstructing the					
		No associated	laryngeal inlet					
		voice change or						
		dyspnea						
Lin *et al*, 2015	37/F	Painless swelling	1×1 cm nodule on	Not mentioned	Further excision	Not mentioned	CKAE1/3 (+),	(17)
		on the ventral	the left ventral		with safe resection		p63 (+), SMA (−),	
		tongue that had	tongue		margin		CD10 (−), S-100 (−),	
		been present for					GFAP (−), MSA (−)	
		months					and *EWSR1* gene	
							rearrangement	
Hu and Li, 2005	69/M	Restricted tongue	5.0 cm maximum	1/64	Surgical excision +	No local	EMA (+), CK8 (+),	(18)
		movement for	diameter at the		postoperative	recurrence or	CK18 (−),	
		5 months	base of the tongue		radiotherapy	metastasis at	CKHMW (−),	
						4-month	CK10/13 (−),	
						follow-up	S-100 (−), SMA (−),	
							and calponin (−)	
Bala-	35/M	Swallowing	3.0×2.0 cm;	Cervical	Excisional biopsy	No local	Immunohisto-	(19)
krishnan *et al*,		difficulty for	smooth and	second abdo-	was performed with	recurrence or	chemistry was not	
2002		5 months and	elevated yellowish-	minal lymph	the assistance of a	metastasis at	performed	
		feeling of choking	white lesion	nodes and	microlaryngoscope	1-year		
		sensation in the	extending from the	submandibular	and surgical	follow-up.		
		throat	midline to the	lymph nodes	microscope; 2 weeks	The patient		
		intermittently	lingual groove of	could be	later, extensive re-	accepted the		
			the right tonsil	palpated on	section of the lesion	result because		
				the right side,	through a trans-	of good		
				each	mandibular	phonetics		
				~2.0×1.0 cm,	approach was			
				with no	performed, with the			
				pressure pain	defect reconstructed			
					with a tongue flap.			
					A scapulohyoid			
					supraglottic cervical			
					lymph node dis-			
					section and frozen-			
					section biopsy of an			
					isolated lymph node			
					were performed			
					prior to the resection			
Chapman *et al*,	68/F	Not mentioned	3.0 cm; the left	Not mentioned	Excisional biopsy	No local	CK5 (+), p63 (+),	(20)
2018			base of the tongue		with positive	recurrence or	S-100 (−), SMA (−),	
					margins. Left level	metastasis at	negative *MAML2*	
					II–III lymph node	18-month	breakage, positive	
					clearance showed	follow-up	*EWSR1* rearrange-	
					negative results.		ment and intact *ATF1*	
Wang *et al*, 2018	69/M	Not mentioned	The base of the	1/64	Combined left	No local	CK (+), S-100 (+)	(21)
			left tongue		lingual and cervical	recurrence or	and SMA (−)	
					curettage + right	metastasis at		
					zonal cervical	42-month		
					lymph node	follow-up		
					dissection +			
					pectoralis major			
					myocutaneous flap			
					repair			
Wang *et al*, 2018	47/F	Dysphagia	Base of the left	No metastasis	Extended resection +	No recurrence at	CK (+) and SMA (−)	(21)
			tongue		adjacent flap repair	12-month		
					without cervical	follow-up		
					lymph node			
					dissection			
Wang *et al*, 2018	67/M	Not mentioned	The base of the	0/17	Extended resection +	No recurrence	CK (+), SMA (−)	(21)
			right tongue		right zonal cervical	at 6-month	and S-100 (−)	
					lymph node	follow-up		
					dissection + radial			
					forearm free flap			
Al Zadjali *et al*,	38/F	A 2-week history	2.9×5.2×3.2 cm;	2/32	Tracheostomy +	No recurrence	CK5 (+), CK7 (+),	(22)
2023		of a sore throat	left root of the		Transcer-vical	at 12-month	p40 (+), p63 (+),	
		superimposed on	tongue and left		transmandibular	follow-up	S-100 (−),	
		a 4-year history	tonsil		approach for wide		SOX10 (−) and	
		of hemoptysis.			excision of the		*EWSR1-ATF1* fusion	
		During this time,			lesion + neck			
		they also			dissection + radial			
		experienced			forearm free flap +			
		progressive			postoperative			
		dysphagia and			adjuvant			
		odynophagia			radiotherapy			
O'Sullivan-	59/F	Dysphagia	3 cm; left root of	No metastasis	Surgical extended	No recurrence	CK (+), p63 (+),	(23)
Mejia *et al.* 2009			the tongue.		resection	during	EMA (+), PAS (+),	
						follow-up	CAM5.2 (weak +),	
							S-100 (−), desmin (−),	
							TGB (−) and Mu (−)	
Suzuki *et al*, 2006	66/F	Dysphagia, denial	4×3×2.5 cm;	No metastasis	Tracheostomy +	No recurrence	Not mentioned	(24)
		of respiratory	tongue root		resection via the	at 21-month		
		distress			paramedian	follow-up		
					mandibulotomy			
					combined with a			
					right-sided supra-			
					omohyoid neck			
					dissection. A macro-			
					scopic surgical			
					margin was set at			
					~10 mm. Both the			
					lingual and			
					hypoglossal			
					nerves were			
					preserved.			
Zhao *et al*, 2022	67/M	Neck mass found	The right root of	Right cervical	Extensive total	No recurrence	CK5/6 (+), p40 (+),	(25)
		for >1 year	the tongue	lymph node	excision of the	at 26-month	p63 (+), CK7 (+),	
				metastasis	mass + cervical	follow-up	EMA (+),	
				(3/16)	lymph node		Ki-67 (5-10% +),	
					dissection		CD117 (−),	
							CD10 (−), GFAP (−),	
							SMA (−), S-100 (−),	
							calponin (−), positive	
							breakage recom-	
							bination of *EWSR1*	

CK, cytokeratin; HCCC, hyalinizing clear cell carcinoma; SMA, smooth muscle actin; EMA, epithelial membrane antigen; CAM5.2, cytokeratin antibody marker 5.2; GFAP, glial fibrillary acidic protein; PAS, periodic acid-schiff.

## Data Availability

The data generated in the present study may be requested from the corresponding author.

## Abbreviations

CT: computed tomography
FISH: fluorescence *in situ* hybridization
HCCC: hyalinizing clear cell carcinoma
WHO: World Health Organization
