# Supplementary material for: A rare case of hyalinizing clear cell carcinoma of the tongue root: A case report and literature review
Source: Oncol Lett. 2025 Feb 4;29(4):168. doi: 10.3892/ol.2025.14914 (PMC11826299; doi:10.3892/ol.2025.14914)

Figure S1. Fluorescence *in situ* hybridization assay results for *EWSR1* gene analysis. DAPI, blue; probe, red and green. Results of 100 cells counted: 14 cells showed only fusion signals and were classified as negative signal cells (14%); 48 cells exhibited distinct red and green signals without fusion, identified as positive signal cells (48%); six cells showed a combination of red signals with fusion signals, identified as negative signal cells (6%); nine cells displayed green signals with fusion signals, identified as negative signal cells (9%); 23 cells showed fusion signals along with red and green probe signals, identified as positive signal cells (23%). These results suggested the presence of the *EWSR1* gene. Magnification, x500.

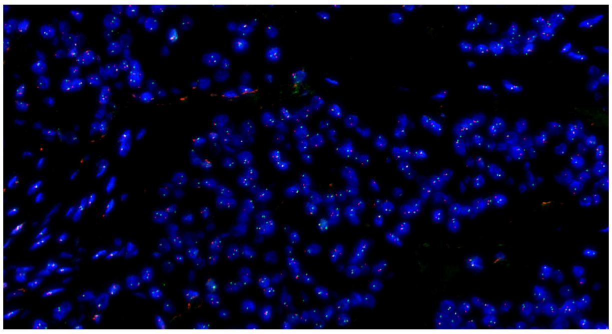

Supplement: Supporting Data [file Supplementary_Data.pdf]
